# Case Report: Acute lead poisoning from bullet ingestion in a captive cheetah (*Acinonyx jubatus*) in Namibia: implications for wildlife management

**DOI:** 10.3389/fvets.2025.1576760

**Published:** 2025-05-09

**Authors:** Catherine Hauw, Anne Schmidt-Küntzel, Ana F. Basto, John Yabe, Niall McCann, María Díez-León, Laurie Marker

**Affiliations:** ^1^Royal Veterinary College, University of London, London, United Kingdom; ^2^Institute of Zoology, Zoological Society of London, London, United Kingdom; ^3^Cheetah Conservation Fund, Otjiwarongo, Namibia; ^4^School of Veterinary Medicine, University of Namibia, Windhoek, Namibia; ^5^Defend, London, United Kingdom

**Keywords:** acute lead poisoning, lead ammunition, cheetah (*Acinonyx jubatus*), leopard (*Panthera pardus*), dietary lead exposure, wildlife management, bone lead concentration (BLC), meat contamination

## Abstract

The case study describes a suspected instance of lead poisoning in a cheetah in Namibia. While lead toxicity is well-documented in carnivorous birds, this case study is only the second of two publications on lead poisoning in cheetahs. Furthermore, we included the first video documentation of acute lead poisoning in this species, showcasing neurological signs of heightened excitability, arched back, raised tail, and excessive salivation. The cheetah died within 48h of first report of clinical signs and post-mortem examinations revealed a bullet in the cheetah's stomach and extensive organ necrosis with multifocal hemorrhages in hepatic and renal tissues. The ingestion of the bullet likely resulted from the feeding of a game-meat carcass which was hunted with lead-ammunition. The cheetah's liver and kidney samples contained lead levels of 38.25 and 56.03 mg/kg dry weight, respectively, consistent with acute lead poisoning. Furthermore, bone lead was shown to be of 1.44 mg/kg dry weight suggesting additional non-lethal chronic exposure. This case highlights the challenges of wildlife management associated with lead ammunition use, particularly in the context of carcass feeding, a common practice for captive carnivores in southern Africa.

## Introduction

Lead (Pb) exposure has garnered worldwide attention for many years, presenting a One Health concern due to its harmful impact on ecosystems, animals, and humans (1). Hunting with lead ammunition has been identified as a primary source of lead exposure for wildlife (2). In the context of captive carnivores such as cheetahs (*Acinonyx jubatus*) in southern Africa, exposure to lead ammunition can occur via ingestion of meat obtained from animals that have been hunted for food or sport or slaughtered for consumption using lead ammunition. Cheetahs are of particular interest due to the species' conservation significance, classified as endangered in Namibia (3). At the time of writing, there were only an estimated 6,517 adults and adolescents left in the wild worldwide (4), and over 500 kept in captivity in southern Africa [of which 486 reported in the International Studbook (5)], where they are one of the most frequently encountered species of captive carnivore. Up to this point, only one publication has documented instances of lead poisoning in cheetahs. North et al. (6) reported suspected cases in two captive cheetahs in South Africa, both of which were regularly fed hunted game birds or antelope. This case report represents the second documented instance of acute lead poisoning in cheetahs, suggesting that this health issue may be more relevant to cheetah health and conservation than what could be assumed based on one single publication.

Lead is widely acknowledged as an extremely toxic metal and unlike several other trace metals like iron or manganese, lead does not serve any physiological purpose (7). There is no recognized safe threshold for lead exposure, and even minimal intake of lead has the potential to be detrimental to both human and animal health (8).

Lead poisoning can present itself in either acute or chronic forms. Acute intoxication typically occurs as a result of animals ingesting lead bullets or pellets. Acute lead toxicity leads to neurological, gastrointestinal, and cardiovascular signs, with the potential of causing extensive damage to organs and ultimately results in fatality (9). Chronic intoxication typically occurs as a resulting of repeated ingestion of smaller amounts of lead, such as through feeding of meat contaminated by lead scatter originating from the fragmentation of the bullet upon impact (10). Gradual build-up of lead in tissues resulting from consistent consumption of lead-contaminated meat also gives rise to chronic systemic effects, diminishing the overall wellbeing of the animals (10).

## Case description

### Clinical observations

On the evening of the 8th of August 2021, a three-year-old female cheetah (NA-AJU1899 “Adina”), temporarily kept in a holding boma at a private nature reserve in Namibia, exhibited mild nonspecific changes in behavior, qualified as “acting odd” by observers. Two days earlier, at the time of the previous routine feeding, the animal appeared to be in good health, bright, and alert. The female was part of a rehabilitation program to provide cheetahs with an opportunity to return to the wild (11). She had been brought to the Cheetah Conservation Fund (CCF) rescue center at eight months of age with her male sibling and was raised with minimal human interaction in preparation for relocation and release (11). As part of the rehabilitation programme (11), the female was fed whole game meat twice a week in the months leading up to her release, to maximize her chances of survival by habituating her to feed on carcasses of species she would typically hunt in the wild. This part of the rehabilitation process took place in a large pre-release enclosure (i.e., 100 ha boma) located in the 65,000 ha reserve into which the female was intended to be released. It is during this time in the pre-release enclosure that the reported health issues were observed. Similar holding conditions are also commonly adopted as part of meta population management of large carnivores (12).

**Figure 1:**
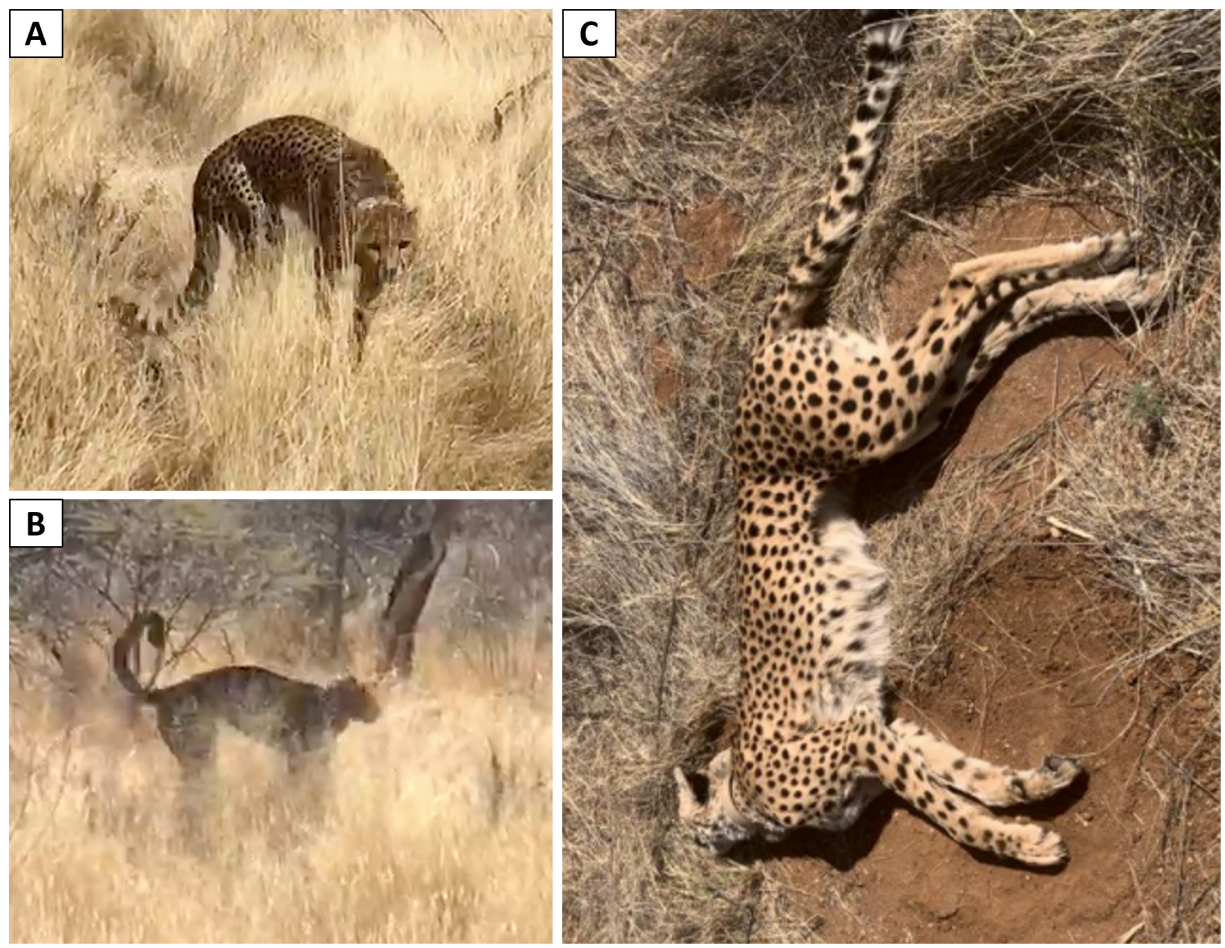
Cheetah displaying ataxia associated with **(A)** arched back and **(B)** raised tail on the first day of clearly identifiable signs (morning after non-specific change in demeanor). **(C)** Cheetah at time of death showing signs of convulsion (opisthotonos) with evidence of seizure activity prior to death.

The morning after the initial, non-specific signs were first observed, the cheetah's condition had deteriorated, displaying increased sensitivity to external stimuli and worsening ataxia and disorientation, with an arched back (kyphotic posture) (Figure 1A) and a raised tail (Figure 1B). The scheduled feeding attempt failed due to uncharacteristic lack of interest and hyperexcitability of the animal. The CCF veterinary team was called to assess the health status of the cheetah. Efforts to approach the animal were unsuccessful due to the animal's heightened alertness. By the following morning, visual inspection indicated that neurological signs (both motor neural signs and sensitivity to external stimuli) had drastically worsened, and also included involuntary movements. Darting was impossible due to the absence of a clear line of sight at a distance of 100 m and the unpredictable behavior of the animal (Supplementary Video). Approximately 2 h later (within 48 h of displaying the first signs of changed behavior), the cheetah was found deceased, with signs of paddling compatible with seizures (Figure 1C), as well as hypersalivation.

### Post-mortem findings

The carcass was sent to CCF for a post-mortem examination. The animal was in good body condition (Body Condition Score = 5/9), supporting an acute disease progression. No external wounds or evidence of snake bite were found. Saliva around the mouth indicated pre-mortem sialorrhea. Postmortem blood samples could not be obtained, precluding hematological analyses. Rabies was eliminated from the differential diagnosis as brain tissue tested negative for rabies (Pathcare Laboratory, Namibia).

**Figure 2:**
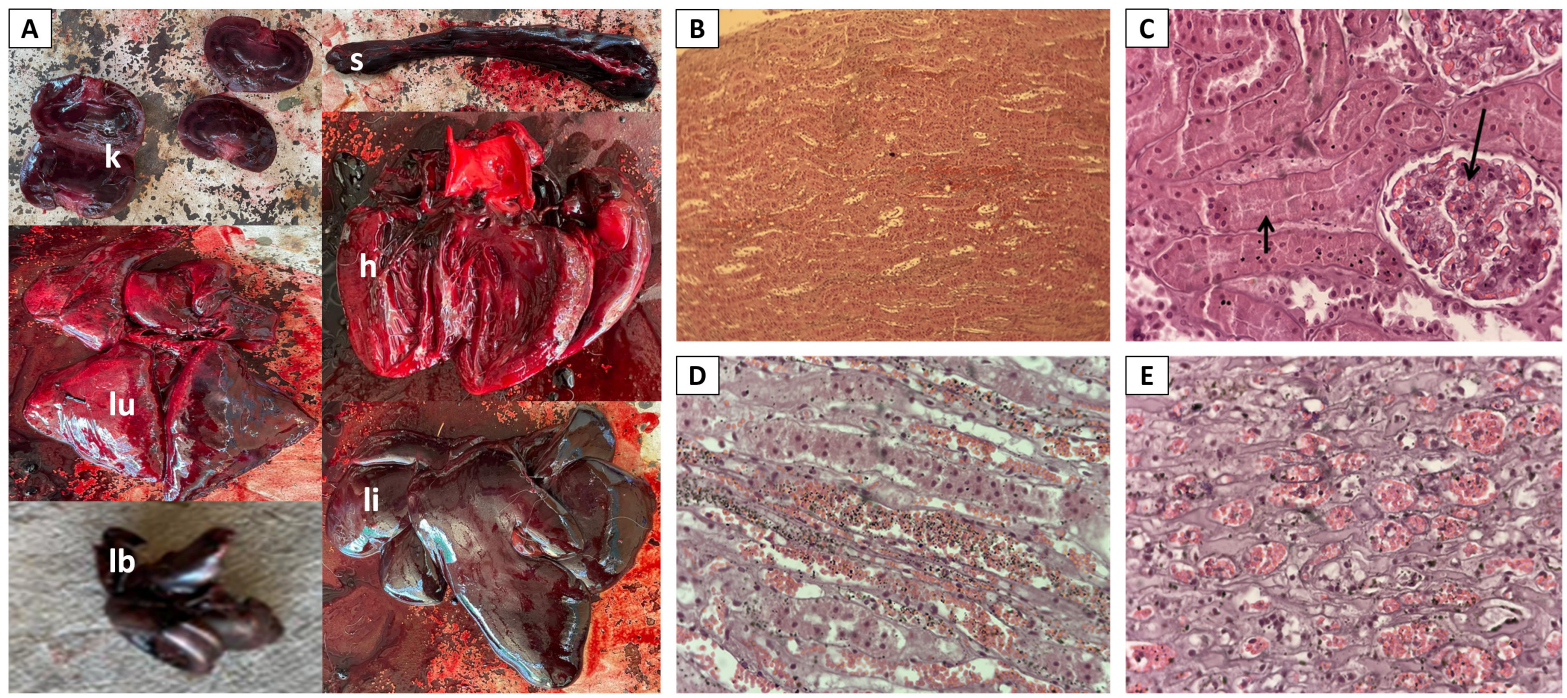
Post-mortem lesions in a cheetah that died of acute lead poisoning. **(A)** Hemorrhages and congestion in organs: kidneys (k), lungs (lu), spleen (s), heart (h), liver (li), and chewed lead bullet found in stomach (lb). **(B–E)**: Histopathology showing typical lesions of acute tubular necrosis. **(B)** Extensive hemorrhages in the tubules (×4). **(C)** Necrosis of the tubules showing loss of tubular epithelial cells (short arrow) and hemorrhages in the glomeruli (long arrow) (×40). Marked degeneration and necrosis accompanied by hemorrhages in **(D)** proximal and **(E)** distal tubules (×10).

The liver, spleen, kidneys, heart, lungs, and intestines were congested and showed widespread vascular damage and hemorrhages. The liver was markedly enlarged and congested, with a dark reddish-black discoloration, friable texture, and subcapsular hemorrhages throughout; the spleen had similar pathological findings, consistent with acute splenomegaly from hypovolemic shock and endothelial dysfunction. Both kidneys exhibited a loss of corticomedullary differentiation, and cross-sections revealed diffuse hemorrhagic infiltration and structural disruption, indicative of acute tubular necrosis which was confirmed in the histopathology. The heart was diffusely congested, with multifocal hemorrhages throughout the ventricular walls and epicardium, and appeared enlarged, suggesting acute cardiomyopathy. The lungs were heavy, congested, and oedematous, with diffuse hemorrhages and foam-filled airways (Figure 2A). Upon opening the abdominal cavity, ~1 liter of free blood was present in the peritoneum, with an additional 500 ml in the thoracic cavity, indicative of severe internal hemorrhage. Brain-related modifications could not be assessed since the head had been sent away for rabies testing.

While these macroscopic findings were non-specific and non-diagnostic, the presence of a lead bullet of caliber 0.308 Winchester and weight 110 grains (7.1 grams) in the stomach suggested lead intoxication as potential cause. Lead analysis was performed in fresh kidney tissue by the Central Veterinary Laboratory of Namibia, using AAS-Graphite furnace. The analysis yielded high concentrations of lead (62.98 mg/kg dry weight, dw) in the cheetah's liver.

Two years post-mortem, lead analysis was repeated as part of a broader study aiming to assess lead levels in cheetahs (Hauw et al., in review), using formalin-fixed liver and kidney tissue, as well as a tibia bone sample. Following nitric acid-digestion, lead was measured with Inductively Coupled Plasma Mass Spectrometry (ICP-MS) at Hokkaido University, Japan (see Hauw et al., in review, for details on the laboratory analysis). The concentrations of lead in the liver and kidney tissues were high (38.25 mg/kg dw and 56.03 mg/kg dw, respectively), while bone lead levels were moderate (1.44 mg/kg dw).

Formalin-fixed liver and kidney samples were routinely processed and stained with haematoxylin and eosin for histopathology. Histopathological examination of the kidneys revealed no renal intranuclear inclusions typical of lead toxicity; however, kidneys showed extensive degeneration and necrosis of the tubules (Figures 2B–E), and marked hemorrhages were seen in the glomeruli, tubules and interstitium (Figures 2B–E), providing valuable evidence for the diagnosis of lead exposure (13). In the liver, extensive necrosis was seen in the hepatocytes, effacing the architecture of the tissue (data not shown).

## Discussion

The diagnosis of lead poisoning is strongly supported by the elevated concentrations of lead in the liver and kidneys, exceeding 10 mg/kg (14). This etiology is further corroborated by neurological clinical signs, gross pathological lesions in post-mortem, histological findings, and ultimately, the presence of a lead bullet in the cheetah's stomach.

There are clear similarities between the present case and those reported by North et al. (6), such as raised tail, arched back, excessive salivation, seizures, and rapid death. Although the seizures in this case were inferred from the disturbance signs observed at the mortality site and could not be directly observed due to the pre-release layout. A distinct clinical finding in the current case was the presence of congestion and hemorrhages across multiple organs. While hemorrhage is not a common symptom of lead toxicosis, lead toxicosis is known to cause increased oxidative stress, which in turn disrupts endothelial function, induces cytotoxicity, and heightens vascular permeability, and thereby predisposes to hemorrhage (15, 16).

While a diagnosis of lead poisoning could be established with near certainty, it is important to remain open to the possibility that other factors may also have contributed to some of the lesions and clinical signs. Other toxicants were considered but deemed unlikely given the controlled captive environment. Hypovitaminosis A, another possible cause of neurological signs, was ruled out as the animals were consistently fed supplemented meat, ensuring adequate intake of necessary nutrients and vitamins. Snake bites involving haemotoxic venom (such as from a boomslang), were considered unlikely as no evidence of envenomation or bite marks was observed during the post-mortem examination. Furthermore, there was no evidence of degenerative, infectious, traumatic, or parasitic disease: rabies was ruled out through diagnostic testing; infection could not be verified with hematology as no blood sample was available, however no signs of infection were found on the carcass and no clinical signs indicative of fever were observed prior to death.

The ingestion of a lead bullet is the most probable cause, with the bullet likely having remained in the stomach for several days due to gastrointestinal stasis from lead poisoning. Prolonged gastric retention, combined with the acidic environment of the stomach, would have facilitated the dissolution and absorption of lead, elevating blood lead levels to a fatal threshold (17, 18).

Kidneys are reliable indicators of recent lead exposure and are particularly sensitive tissues prone to heavy metal toxicity (19). This heightened retention of heavy metals in the kidneys can further exacerbate the damage caused by lead exposure (20). Kidney lead concentrations exceeding 15 mg/kg dw were linked to both structural and functional kidney impairment; kidney lead levels surpassing 80 mg/kg dw were associated with adverse outcomes such as weight loss and mortality in humans (18). As opposed to North et al. (6), which report lead levels above 80 mg/kg dw [101.4 mg/kg dw, derived from wet weight using a conversion factor of 1/6.5 for kidney (15)], this case study found that the values at the time of death in cheetahs only reached 56.03 mg/kg dw (62.98 mg/kg dw in fresh tissue analyzed in 2021), suggesting that a lower threshold may be sufficient for acute lead poisoning in cheetahs. Liver lead concentrations exceeding 14.4–40 mg/kg dw are commonly considered indicative of lead toxicosis in domestic cats (21). Here too, the values of this case (38.35 mg/kg dw), were elevated, yet lower than those reported by North et al. (6); 68.0 mg/kg dw, derived from wet weight using a conversion factor of 1/4.0 for liver (16).

The moderately elevated bone lead concentration (BLC 1.44 mg/kg dw) observed in the cheetah's tibia reflects cumulative lifetime exposure, as bone lead serves as a long-term reservoir for lead accumulation (22). Acute exposure, such as the ingestion of the bullet found in the stomach, would not yet be expected to have had a significant impact on BLC, as it typically takes several weeks for lead to transfer from the bloodstream to other soft tissues and eventually to bone compartments (23). Therefore, BLC is more likely to reflect chronic, long-term exposure rather than a single acute event. This chronic bone accumulation probably resulted from the consumption of meat contaminated with lead bullet fragments during routine feeding in captivity or in the pre-release boma. The ultimately fatal acute lead exposure of this cheetah shortly prior to release is on the other hand likely linked to the ingestion of a lead bullet through the consumption of hunted game. Therefore, this cheetah was likely affected by both, chronic and acute exposure from feeding, underscoring the risk to captive predators associated with the use of lead ammunition in wildlife-rich environments and conservation sanctuaries.

The present case study underscores the pressing necessity to shift from lead ammunition to non-lead alternatives, not only for hunting sports but also for food preparation within the realm of wildlife conservation sanctuaries, which serve a vital function in safeguarding and rehabilitating wildlife. The use of lead bullets within these sanctuaries can run counter to their mission as this practice unnecessarily causes health risks, potentially impeding conservation initiatives. Similarly, in South Africa, managed metapopulation strategies to conserve endangered animals often involve bomas, where animals are fed entire carcasses shot with lead bullets before being released into the protected wild. Non-lead ammunition, such as copper or steel bullets, have been developed as a safer alternative. These alternatives are just as effective as lead bullets for hunting and shooting sports, without the associated risk of lead contamination in the environment and food chain. When non-lead options are unavailable, the shot should be aimed at the animal's head so the lead bullet can be safely removed by sectioning the neck If this is not possible, a margin of about 40 cm of surrounding meat should be removed to account for lead fragmentation in adjacent tissue. By adopting non-lead ammunition, we can significantly reduce the risk of lead toxicity and its devastating consequences in both captive and wild animals, and the wider environment.

## Data Availability

The original contributions presented in the study are included in the article/Supplementary material, further inquiries can be directed to the corresponding authors.
